# ‘They will be afraid to touch you’: LGBTI people and sex workers' experiences of accessing healthcare in Zimbabwe—an in-depth qualitative study

**DOI:** 10.1136/bmjgh-2016-000168

**Published:** 2017-04-13

**Authors:** Jennifer Hunt, Katherine Bristowe, Sybille Chidyamatare, Richard Harding

**Affiliations:** King's College London, Cicely Saunders Institute, Department of Palliative Care, Policy & Rehabilitation, London, UK

## Abstract

**Objectives:**

To examine experiences of key populations (lesbian, gay, bisexual, trans and intersex (LGBTI) people, men who have sex with men (MSM) and sex workers) in Zimbabwe regarding access to, and experiences of, healthcare.

**Design:**

Qualitative study using in-depth interviews and focus groups, with thematic analysis.

**Participants:**

Sixty individuals from key populations in Zimbabwe.

**Setting:**

Participants were recruited from four locations (Harare, Bulawayo, Mutare, Beitbridge/Masvingo).

**Results:**

Participants described considerable unmet needs and barriers to accessing basic healthcare due to discrimination regarding key population status, exacerbated by the sociopolitical/legal environment. Three main themes emerged: (1) key populations' illnesses were caused by their behaviour; (2) equal access to healthcare is conditional on key populations conforming to ‘sexual norms’ and (3) perceptions that healthcare workers were ill-informed about key populations, and that professionals' personal attitudes affected care delivery. Participants felt unable to discuss their key population status with healthcare workers. Their healthcare needs were expected to be met almost entirely by their own communities.

**Conclusions:**

This is one of very few studies of healthcare access beyond HIV for key populations in Africa. Discrimination towards key populations discourages early diagnosis, limits access to healthcare/treatment and increases risk of transmission of infectious diseases. Key populations experience unnecessary suffering from untreated conditions, exclusion from healthcare and extreme psychological distress. Education is needed to reduce stigma and enhance sensitive clinical interviewing skills. Clinical and public health implications of discrimination in healthcare must be addressed through evidence-based interventions for professionals, particularly in contexts with sociopolitical/legal barriers to equality.

**Key questions**

**What is already known about this topic?**

- ‘Key populations’ are disproportionately affected by serious illnesses, including cancer and HIV.
- However, they have significantly lower uptake of essential health services due to marginalisation, stigma and human rights violations.
- Very few studies have considered access to health services for key populations in low-income and middle-income countries, beyond the context of HIV.

**What are the new findings?**

- Participants described barriers to accessing even basic healthcare due to discrimination perpetrated by healthcare professionals.
- Equal access to care was dependent on conforming to ‘sexual norms’.
- Healthcare professionals’ personal attitudes affected care delivery, and key populations were perceived to have brought illnesses on themselves through sexual behaviour.

**Recommendations for policy**

- Key populations experience unnecessary suffering from untreated conditions, exclusion from healthcare and extreme psychological distress.
- There is a need for safe confidential environments, cultural sensitivity training and public health strategies to reduce stigma and improve and increase access to healthcare for key populations.
- Policy must enshrine non-discrimination for key populations with respect to access to public services.
- Professional communication skills to enable patients to share key aspects of identity and behaviour must be combined with enforcement of professional standards that require antidiscriminatory practice.

## Introduction

‘Key populations’ describe individuals who are disproportionately affected by some serious illnesses (such as HIV), but have significantly lower uptake of essential health services due to social marginalisation, legal and social conditions, stigma and human rights violations.^1^ The term is especially relevant in determining an appropriate response to the HIV epidemic where inequities in vulnerability are experienced by different subgroups within the population. Key affected populations include lesbian, gay, bisexual, trans and intersex (LGBTI) people, including men who have sex with men (MSM) and women who have sex with women (WSW), sex workers (people who exchange sexual services or favours for money or gifts) and injecting drug users (IDU). MSM and WSW have been consistently used as terminology to include people globally, and within Africa (including Zimbabwe), who may not identify with ‘gay’ or ‘lesbian’.^2^^–^4

Key populations may experience health vulnerability beyond the risk of HIV infection. There is increasing recognition that LGBTI people represent minority communities with unique healthcare needs.^5^ ^6^ Access to health and psychosocial care for marginalised populations in general, and in Africa in particular, is poorer than for the general population. LGBTI people have a relatively higher prevalence of life-limiting illnesses, particularly cancer,^7^ ^8^ and greater all-cause mortality than heterosexual people.^9^ Discrimination against any minority or socially disadvantaged group is a significant risk factor for stroke, heart disease, poor mental health, psychological distress and depression.^10^ ^11^ There is global variance in acceptance of homosexuality with secular and more affluent countries demonstrating greater acceptance, and widespread rejection in Africa and in poor, highly religious countries.^12^

Estrangement from family and stigmatisation from healthcare staff reinforce widespread discrimination against key populations.^13^ Globally, MSM are disproportionately affected by HIV. In sub-Saharan Africa, MSM have an HIV prevalence four times that of heterosexual men.^14^ Stigma and discrimination against LGBTI individuals are common in Southern Africa,^15^ and same-sex practicing Africans living with HIV are known to be marginalised by HIV programmes, increasing the probability of premature death.^16^ Research in Central and Southern Africa has found that WSW have poorer sexual and reproductive health^17^ and higher prevalence of forced sex,^18^ while MSM have experience of human rights abuse.^19^ Perceptions of stigma are known to discourage people from testing and seeking treatment worldwide,^20^ and discriminatory practices often result in exclusion and inadequate care provision.^13^

Despite historical acceptance of same-sex relationships in Africa,^21^ the current Zimbabwe Criminal Law (Codification and Reform) Act makes specific sexual acts illegal, but falls short of criminalising LGBTI status.^22^ The popular belief, however, driven by political attitude and an uninformed media is that it is a crime to identify as LGBTI. Homophobic statements by government leaders in public fora and reported in the national press contribute to a misinformed, highly discriminatory sociopolitical environment. While recent moves towards upholding rights of sexual minorities in Malawi have been greeted with cautious optimism,^23^ in several African states, most notably in Nigeria and Burundi, attempts have been made to extend criminalisation of same-sex practice with harsh and sometimes lethal punitive measures. The unintended health-related consequence of such highly stigmatised environments is reluctance by sexual minorities to access early diagnostic and treatment services and care programmes.^14^

Sex workers also routinely experience discrimination, hostility, denial of, or precarious access to, health services across Kenya, Zimbabwe, Uganda and South Africa.^24^ In Zimbabwe, the Criminal Law Act makes soliciting, procuring and living off the earnings of sex work a crime. Research with sex workers in Africa has generally focused on risk behaviours and disease transmission, rather than illness experiences and access to care although health worker stigma has been identified as one of several challenges facing sex workers accessing hospital HIV treatment in Zimbabwe.^13^

There has been limited research of the specific health outcomes and experiences of trans people, outside of the context of HIV and studies of MSM or WSW. However, one study of trans people in South Africa reported unacceptable care, with frequent experiences of hostility and discrimination.^25^ To date, no study has aimed to understand the experiences of access to healthcare (beyond HIV) across key populations in Africa. The aim of this study was to explore the healthcare experiences of key populations (LGBTI people and sex workers) in Zimbabwe regarding formal healthcare access and experience of care received.

## Methods

### Study design

In-depth qualitative interview and focus group study.

### Population

For this study, key populations included sex workers and sexual and gender minorities (LGBTI individuals and MSM). The selection was focused on those socially excluded populations whose sexual behaviours promote higher incidence of infectious and non-communicable life-limiting illness due to risk behaviours which may be linked to discrimination.^26^ In Zimbabwe, LGBTI people, MSM and sex workers are legally and socially marginalised. To access these ‘hard to reach’ populations, organisational leaders of agencies working with key populations were asked to identify and recruit participants in line with purposive sampling criteria. A traditional approach of recruiting via health centres and clinics was considered unrealistic given the sociopolitical context. A summary of the approved project was attached with recruitment guidelines, including a request for one participant (this determined by time and financial constraints) at each site to volunteer to be interviewed separately to explore personal narratives pertaining to illness in key populations in more depth.

### Ethical considerations

Ethical approval for the study was obtained from the Medical Research Council of Zimbabwe (reference number MRCZ/A/1881). Recruitment was undertaken with clear safety protocols to minimise risk to participants and researchers. A distress protocol was developed in case any interviewee becomes distressed, fatigued or unable to continue for other reasons, and a representative from a relevant support organisation was available to address any adverse events. Interviews were conducted in places considered safe by the relevant organisation. Before starting the interview/focus group and having received an explanation of the study, each participant signed a consent to record and a separate participation consent form. Confidentiality was ensured throughout the research process.

### Participants

Participants were purposively sampled by age (aged at least 18 years), gender, sexual orientation, gender identity and sex worker status from four centres in Zimbabwe. The sample was drawn from those living in the two main urban areas as well as smaller centres near border posts, mines and truck stops known to attract high-risk populations. A Focus Group Discussion (FGD) for sex workers and another for LGBTI was conducted in each of the four centres (eight FGDs) using an open-ended semistructured topic guide. Owing to small-town demographics, Beitbridge and the nearby town of Masvingo were combined as one centre. FGDs ranged from 5 to 10 participants. In response to our request for one volunteer at each site for in-depth interview, we conducted three further interviews with FGD participants: one sex worker in Mutare, one sex worker in Beitbridge and one lesbian woman in Masvingo.

### Interviews/focus groups

The topic guides (see online supplementary appendices 1–4) were shaped by: the study objectives; review of the literature on LGBTI and sex worker healthcare needs and experiences and two pilot FGDs (data not included). The broad areas of enquiry were: illness history and experiences of accessing healthcare; exploration of sexual identity in consultations; communication and sexual identity and involvement of partner/significant others and support structures.

10.1136/bmjgh-2016-000168.supp1 supplementary appendix

10.1136/bmjgh-2016-000168.supp2 supplementary appendix

10.1136/bmjgh-2016-000168.supp3 supplementary appendix

10.1136/bmjgh-2016-000168.supp4 supplementary appendix

All interviews and focus groups were audio recorded. Interviews and focus groups were conducted primarily in English with questions translated into the vernacular by the research assistant as required. The lead researcher (JH) is Zimbabwean, fluent in English and has limited fluency in Shona; the research assistant (SC) is Zimbabwean and fluent in all three main languages (English, Shona and Ndebele). English is the official language of Zimbabwe but was sometimes the second or third language of the participants. Topic guides were translated from English into both vernacular languages.

### Analysis

Data were transcribed verbatim into English by the trilingual research assistant and analysed using thematic analysis^27^ which has five key stages: familiarisation, coding, theme development, defining themes and reporting. Analysis was led by JH. The first stage of analysis was familiarisation where the researcher(s) read, reread and annotated the transcripts alongside research questions. Transcripts were then coded line by line deductively within the core areas of the interview schedules. Subsequently, a coding frame was developed to demonstrate emergent patterns and themes. The coding frame and transcripts of three interviews/focus groups were reviewed by the research team (JH, KB, RH). After discussion (JH, KB, RH), themes were developed and the coding frame revised and applied to the full data set. During subsequent analysis, the themes were developed further and refined with particular attention paid to non-confirmatory cases where emerging themes contradicted more common ideas. Additional themes not captured in the core areas were also noted during this stage of analysis and added to the thematic compilation. Themes were defined and finalised through discussion (JH, KB, RH), and all researchers agreed the final analysis, interpretation and reporting.

## Results

### Participants

Sixty individuals from key populations were recruited for interviews and focus groups from across Zimbabwe (see table 1): Harare (n=17); Bulawayo (n=16); Mutare (n=15) and Beitrbridge/Masvingo (n=12).

**Table 1:** Participants

Focus group and interview participants	60
Site	
Harare	17
Bulawayo	16
Mutare	15
Beitbridge/Masvingo	12
Age (years)	
<35	32
35 and over	27
Information not provided	1
Self-identified gender	
Male	18
Female	42
Ethnicity	
Shona	39
Ndebele	16
Other	5
Self-identified sexual or gender identity/sex worker	
Lesbian	6
Gay	15
Bisexual	3
Trans	2
Intersex	1
Sex worker	33

Thirty-nine participants were of Shona ethnicity, 16 Ndebele and 5 other ethnicity. Forty-two participants were women and 18 men. Just over half (n=32) were <35 years old, and the remainder were 35 or over (n=27, one individual did not disclose their age). Participants self-identified as: lesbian (n=6); gay (n=15); bisexual (n=3); trans (n=2); intersex (n=1) and sex workers (n=33).

### Findings

Three distinct main themes emerged from the data: (1) illnesses have been caused by ‘bad behaviour’ and deserve blame, discouraging key populations from seeking health support: key populations were perceived to have brought illnesses on themselves through sexual behaviour; (2) equal access to healthcare is conditional on conforming to sexual norms: to receive the same access to health and palliative care services as the general population, key populations believed they must pretend, deny or lie about their sexual identity/behaviour and (3) perceptions that healthcare workers were ill-informed about needs of key populations, and personal attitudes impacted on their care delivery: the lack of understanding of, and disrespect for, key populations by health workers resulted in experiences of poor support and provision of care during chronic illness, significantly increasing morbidity and mortality. Themes and subthemes are displayed in table 2. Example quotes are presented in the descriptions below to support each theme.

**Table 2:** Emergent themes and subthemes

Themes	Subthemes
1. Illnesses have been caused by ‘bad behaviour’ and deserve blame, discouraging key populations from seeking health support	Getting what they deserve
	Manifestations of stigma and discrimination
2. Equal access to healthcare is conditional on conforming to sexual norms	Assumptions made about sexuality or sexual practices make them invisible
	If you do not fit you do not get care
3. Perceptions that healthcare workers were ill-informed about needs of key populations, and personal attitudes impacted on their care delivery	Compromised professional medical standards, ethics of care and accountability Ignorance, religious views and personal opinions

### Illnesses have been caused by ‘bad behaviour’

#### Getting what they deserve

Participants experienced widespread stigma and discrimination irrespective of their symptoms and illnesses. They described being blamed for their illness as a result of their identity or profession. The discrimination for sexual minorities extended to accusations of witchcraft to explain their condition, bringing a curse on the family or suggestions of divine retribution against their sexual identity.

Healthcare workers had some misconceptions over the whole thing, almost treating it ‘with superstition’ …linking it [prostate cancer] … most likely he is gay… so he wasn't getting the healthcare that he needed to the point that he eventually passed away due to just simple negligence … treating it as some kind of illness that is due to homosexuality.

FGLGH4 Gay man, 25, focus group Harare

For some, the double stigma of being a member of a key population and living with HIV, both considered socially unacceptable, presented a dilemma with participants choosing to disclose one or the other to minimise the risk of negative reactions.

The only time he eventually said he is actually sick [with HIV] was when he was really sick because that is how judgmental the family is. Even if he was heterosexual. So if it's hard for a heterosexual in my family to come out to say that I have this, what more of a person who is gay. They would kick you to the kerb.

FGLGH4 Gay man, 25, focus group Harare

For sex workers, there was evidence that they were reluctantly accepted as an inevitable part of society. However, they were regularly exposed to extremely negative, discriminatory and hostile reactions from health workers.

They can tell you to wait outside and that you want me to touch your rubbish and you are the ones destroying our marriages.

FGSWM8 Sex worker female, 30, focus group Mutare

#### Manifestations of stigma and discrimination

Participants reported humiliating responses and inadequate care from health workers when sexual orientation or sex worker status was disclosed, often resulting in unwillingness to pursue treatment.

If you go to the clinic seeking treatment as a sex worker the way they handle you is unpleasant even if you are explaining your problem nicely. Secondly for medication you might be given Paracetamol when they have the proper medication because they would be fixing us. They say we are doing something illegal which is prostitution so you won't get the medication. So they won't give you the medication when they have it.

FGSWM9 Sex worker female, 50, focus group Mutare

Participants described assessing the level of discrimination of the health worker from their manner and language, which often discouraged disclosure. Fears regarding potential reactions from healthcare professionals resulted in failure to attend the hospital for diagnosis.

I won't go to a hospital and say I have STI. I would rather pay a doctor and get prescribed the amoxicillin or these cheap tablets instead of telling the doctor that I have an STI which I got from my boyfriend. I won't tell him that.

FGLGB5 Gay man, 30, focus group Bulawayo

When patients did disclose their sexual orientation, attention was often diverted from health needs to inappropriate curiosity about their sexual behaviour.

We said … we are a couple. She panicked and she left. The next thing they came. Two of them. And we said yes we are a couple, we are partners and she started asking who the man in the relationship is? How do you do it? You know it just made us uncomfortable.

FGLGH8 Lesbian woman, 41, focus group Harare

The pejorative attitude of health workers led to individuals avoiding seeking healthcare.

I was ignored at the hospital because I had evidence to show that I am a sex worker which was my referral from [sex worker clinic]. They ignored me then said I should pay of which I didn't have any money. So I then went back home and until now I haven't been attended.

FGSWM6 Sex worker female, 31, focus group Mutare

Experiences of stigma, discrimination, financial constraints as well as the dilemma posed by attending STI and HIV clinics with an acceptable partner, commonly resulted in late presentation for diagnosis and treatment.

I went to a pharmacist one time and I wanted [postexposure] prophylaxis … he said he needed a prescription and it was during the weekend and he told me to go to my GP and I was thinking what to tell the GP if I were to go there and how would I explain. So the weekend passed and the 72 hours were up.

FGLGBB4 Gay man, 26, focus group Masvingo

Such delays in attending clinics had significant impacts on the health and well-being of these individuals.

### Access to healthcare conditional on conforming to sexual norms

#### Assumptions made about sexuality or sexual practices make them invisible

Communication about sexual identity was limited and primarily framed in monogamous heterosexual terms. This left the onus on the patient to disclose ‘non-conformity’, knowing that doing so may change or halt treatment options. Failure to take a sexual history, even when the presenting symptom (HIV, cervical cancer, genital warts, other Sexually Transmitted Infections (STIs)) was related to sex, afforded individuals anonymity, culminating in heteronormative assumptions and ironically access to healthcare without judgement.

When she accompanies me, we say that we are sisters. So she would just have accompanied her sister to get her medication. They would not know what would be going on.

KIIBB2, Lesbian, 40, interview Masvingo

However, hiding sexual practice had many more negative results and disadvantages. Lesbian, gay, trans and intersex people were unspecified in HIV and health support programmes, increasing pressure for them to conceal their sexual identity to access these services. The standard procedures of treating both or all sexual partners for STIs and encouraging sexual partners to be tested together for HIV were especially challenging for key populations. Sex workers unable to produce a partner reported returning home without treatment, while some sexual minorities admitted to recruiting an acceptable proxy partner.

I could not take my girlfriend to the clinic with me and I would take any man on the road to go with me so I could get treated.

FGLGBB5 Lesbian woman, 40, focus group Masvingo

Sex workers described becoming highly mobile to escape identification, jeopardising continuity of healthcare, or concealing their profession to secure treatment.

The nurses there would go about calling you a prostitute in the wards … but I said I was a cross border trader because I was in a ward with better off people so I had to lie of what I did for a living.

FGSWH1 Sex worker female, 36, focus group Harare

Conversely, one participant claimed to be a sex worker to benefit from the free treatment provided at the clinic reserved for sex workers only. There were also reports of concealing symptoms to avoid questions that may lead to disclosure of sexual identity or sex worker status, leading to inaccurate diagnosis, inadequate treatment and often risky self-diagnosis.

Most of the times you hide what you would be feeling like. You have an STI and you end up saying you have a headache and don't get treated properly.

FGSWB7 Sex worker female, 25, focus group Bulawayo

#### If you do not fit, you do not get care

Participants described health workers displaying overt discrimination against key populations, with particularly negative attitudes towards men practising anal sex. In addition, the punitive socioeconomic and legal environment acted as a tangible barrier to accessing care.

When you go [to the clinic] they say you have to report to the police. So their first target is for me to go to the police and say I am gay and I am sick and then get a letter from there and go the clinic. But now it's a matter of you are now a targeted person from the community you see.

FGLGH3 Trans man, 28, focus group Harare

Owing to estrangements from biological family, healthcare was ultimately provided by partners and friends from key populations groups.

They might support me morally but not financially because there's this belief that the LGBTI community infects each other, they are reckless and their multiple partners, that you got it yourself and they will come and visit and bring fruits but for the other problems they say that I should go to my community that I say understands me, they should pay for me.

FGLGB2 Bisexual female, 24, focus group Bulawayo

### Perceptions that healthcare workers were ill-informed about needs of key populations, and personal attitudes impacted on their care delivery

#### Compromised professional medical standards, ethics of care and accountability

Participants living with a diagnosis of advanced STI, HIV diagnosis or any other chronic illness described failing to receive anything other than basic history taking and simple treatments. Lack of knowledge and experience of working with key populations was a key driver of stigma. Health workers appeared unwilling to examine key populations for fear of possible contamination or sexual predation.

They will be afraid to touch you and will act like even your fingers are very sensitive. I don't know. They will think that you are going to respond.

FGLGB4 Lesbian woman, 25, focus group Bulawayo

When examination did occur, participants expected healthcare professionals to make obvious their disdain.

Like if you have sores or warts on the private parts. If she treats you and looks at them without screwing her face and just looking at them with a neutral face that makes me comfortable.

FGSWH6 Sex worker, female, 37, focus group Harare

Reports of breaches of confidentiality were also common, particularly when attending local clinics.

You find that the nurses live in our communities and once you tell her that mostly the news will spread … you will go out with your friends for drinks and walk past them and they will start talking about you coming to the clinic and the reason.

FGLGB2 Bisexual female, 24, focus group Bulawayo

#### Ignorance, religious views and personal opinions

Participants described care being driven by personal attitudes of health workers, often based on religious beliefs. Participants perceived that health workers abused their professional role by promoting personal religious views and that prejudice against sexual minorities was often framed in moral and religious (Christian) terms.

some of them are Christians and they will want to now start preaching to you that this is not right, you should change.

FGLGB3 Lesbian, 42, focus group Bulawayo

Sex workers, however, reported better care experiences from clinics specifically focused on improving sexual reproductive health for sex workers, attributing this to well-trained health workers.

the first thing they ask me is how my work is and if we are getting money. So by that I will then feel comfortable because they know what I do for a living and they will ask if I feel ok inside or I have a problem. (FGSWBB1 Sex worker 41, focus group Beitbridge)

## Discussion

This was the first study undertaken in Zimbabwe to describe health-seeking behaviours and experiences of LGBTI people and contributes to the growing body of local research on sex workers. Evidence of a blame culture towards key populations by family members, health workers and society in general in Zimbabwe was described, closely reflecting stigma research results in India.^28^ Factors emerged hindering uptake of general health services by key populations, most important of which was the experience of stigma and discrimination from health workers. The findings from this study confirm that misconceptions, limited experience and lack of information about key populations drive fear and prejudice. The study demonstrates how a stigmatising environment discourages early presentation, diagnosis and treatment and that an unwillingness to explore and disclose identity and sexual practice results in inaccurate information, hidden symptoms and harmful behaviour (self-treatments or continuing risk behaviours). Most participants acknowledged there were differences in care afforded to the general population and key populations. These findings are in line with a recent large international survey which found implicit preferences for heterosexual people versus gay and lesbian people by heterosexual healthcare providers.^29^ The interview process itself was identified as an intervention, providing recognition and a voice for key populations, as well as identifying barriers to care.

Terminology in national health strategies (Zimbabwe included) refers to the principles of equity, universal healthcare and quality of health for all. However, this study identifies experiences of stigma, discrimination and refusal of care for key populations across Zimbabwe. Policymakers need to use evidence from this study to advocate for specific inclusion of LGBTI people and sex workers as key populations in health strategies as they are developed or revised. Specific health services for sexual minorities, operated along similar lines to the successful sex worker clinics^30^ and clinics for people living with HIV, may well provide a safer, more effective health environment for LGBTI people. However, the sociopolitical threat creates dangers for such an identifiable location and it may be more viable for support agencies to expand their network of selected ‘LGBTI-friendly’ medical practitioners.

At the service level, the need for cultural sensitivity training for healthcare professionals is well recognised^31^ and emphasised by our findings. Increased awareness of, and information about, the healthcare needs and human rights of key populations need to be woven through all health worker trainings and service implementation. For example, history taking forms can be amended to guide health workers in appropriate terminology, exploring sexual behaviours and comprehensively addressing the needs of all patients.

Finally, at a public health level, support agencies need to increase provision of resources relating to self-protection, sexual health and well-being, alongside programmes to empower key populations, and adaptation of successful campaigns addressing similar taboo topics (eg, HIV, circumcision) to demystify healthcare rights and access.

Recommendations to improve the healthcare experience of key populations, developed from our data, may be relevant in other low-income and middle-income countries that do not currently provide civil rights for these key populations.

This study had some limitations. First, despite working with support agencies, only men who identified as gay, not other MSM, were recruited, and these men may have a different experience of healthcare. As only two trans and one intersex persons were included in the sample, it is probable that specific healthcare issues experienced by gender minorities were underdescribed. However, in this context, their recruitment was a positive achievement, and addresses the criticism of many LGBTI studies not actually recruiting any trans or intersex individuals.^32^ Further research with wider representation could contribute to the limited data available on health needs among trans and intersex people in sub-Saharan Africa.

## Conclusion

This is one of relatively few studies to describe access to and experiences of healthcare (beyond HIV and sexual health) of sex workers and sexual minorities (ie, key populations) in Africa. The findings inform recommendations to provide a safe and confidential environment that facilitates disclosure and ensures access to effective appropriate treatments. Educational programmes are needed to raise awareness, dispel myths associated with key populations, reduce stigma and enhance sensitive clinical interviewing skills. Recognition of a broader interpretation of ‘family’ and relationship configurations would ease the isolation of patients and disenfranchised partners, and increase access to care. The global health agenda must continue to ensure that the clinical and public health implications of discrimination in healthcare are addressed through research^33^ and education of clinicians.^34^ This is especially important in contexts that pose particularly strong legal barriers to equality in care such as in many countries in sub-Saharan Africa^21^ and other less affluent regions, particularly where religion more often plays a central role in people's lives.^12^
