# Supplementary material for: ‘They will be afraid to touch you’: LGBTI people and sex workers' experiences of accessing healthcare in Zimbabwe—an in-depth qualitative study
Source: BMJ Glob Health. 2017 Apr 13;2(2):e000168. doi: 10.1136/bmjgh-2016-000168 (PMC5435254; doi:10.1136/bmjgh-2016-000168)
Supplement: supplementary appendix [file bmjgh-2016-000168supp_appendix1.pdf]

APPENDIX 1 - INTERVIEW SCHEDULE FOR LESBIAN WOMEN, GAY MEN, BISEXUAL, TRANSGENDER PEOPLE AND MEN WHO HAVE SEX WITH MEN

Date	_________________________
Interviewer	_________________________
Town/District	_________________________
Organization/Facility/Care setting	_______________________
Venue	_________________________

Demographic information:

Interviewee name	_______________________
Tribe	_______________________
Age	_______________________
Gender	_______________________
Sexual identity	_______________________
Relationship status	_______________________
Primary diagnosis	_______________________
Time since diagnosis	_______________________

Language(s)	_________________________
Time began	_________________________
Time ended	_________________________

Exploration of Sexual Identity:

- As someone who identifies as X, in what ways do you think your experience of your illness might be different to people who identify as heterosexual?
- How about your experience of care, how might that have been different?
- When you are with your health care providers, (doctors, nurses, and other members of the health care team) do any of them know your sexual identity?
    - YES….how?
    - NO….why?
- Is this how you prefer it to be?
- How do you feel about being asked directly about your sexual identity?

- Should you be asked directly by the health care providers, or should it come from you first?
- What kinds of things make it easier/less easy to let health care providers know your sexual identity? (PROMPT: communicative, environmental, societal, institutional)
- Has the gender of the health care provider been relevant in disclosing your sexual identity?

Communication and Sexual Identity:

- When taking your personal history or talking to you during appointments, do health care providers ever refer to your sexual identity? How do they do it….WELL/BADLY?
- Do you feel they respect and understand your identity? How do they demonstrate this?
- Are there phrases or words that the health care providers have used that made you feel more able to talk about your sexual identity?
- How about in terms of the way they talk to you – their manner, body language?
- Do you find the health care providers use the same words to describe sexual identity as you?
- Can you tell me about when the issue of your sexual identity hasn't been handled well?
- Are there phrases or words used by the health care providers that made you feel uncomfortable about sharing your sexual identity?
- Have there been times when their manner or body language has stopped you sharing your identity? How?
- How would you like your sexual identity to be acknowledged and referred to by the health care providers?

Involvement of Partner: (IF THEY HAVE PARTNER/EQUIVALENT)

- Has your partner or family attended appointments with you?
- Do you want them present?
- Have you felt they would be welcome?
- Can you tell me how they are acknowledged by health care providers?
- Have they talked about what the experience was like for them?
- Would you like it to be any different? How?
- Do you feel your partner/family have enough support?
- What forms of additional support would be useful for them?

Support Structures:

- Where do you get your support from? (PROMPT: partner, family, communities, institutional)
- Are they known to your health care team? How/why?
- Who would you say are your main sources of care? (outside of professional services like doctors nurses and social worker). (PROMPT: CAREFULLY PROBE FOR BIOLOGICAL VS CHOSEN FAMILY AND NETWORKS)

For those with current serious illness: Illness History:

- Can you tell me something about your illness? When did you first become ill?
- Can you tell me where you've received care for your illness? (e.g. Primary, secondary, tertiary care)
- How do you learn about updated developments in your illness and its treatment?
- What are your main needs and problems? (PROMPT: physical, psychological, social spiritual)
- How do health providers help you manage any physical, emotional, social or spiritual problems you or your family or colleagues may encounter from a diagnosis of a life-shortening condition?

Planning for Future Care:

- Looking forward, if your health changes and decisions need to be made about how best to care for you, who would you like to be involved?
- Do you think this will be done? Can you see any problems?
- Have you had this discussion with anyone? Would you like to, and with whom?
- Do you have someone who could arrange this?

Reflections and Recommendations:

- When you discuss you, your life, and what matters to you with your health care providers, do you think your sexual identity should be part of that discussion? Why? How?
- Since you have had this diagnosis, do you ever feel that you have been treated differently or unfairly because you identify as X?

- Do you have any questions that you think you'd like to ask your health care providers but don't?
- Are there questions you feel the health care providers don't ask you?
- Has any health provider ever discussed palliative care with you?

The aim of our study is to help make LGBT and MSM people know that they and their partner or significant others can expect good care when they have a serious illness, but also to help health care teams to provide better care.

- What should we teach health care providers?
- What things do you think are important for us to tell the LGBT and MSM communities? (PROMPT: are there barriers or facilitators to care that we need to explore/highlight)
- What do you think would be the best materials / media to share our findings?
- If there was one thing that we could change, what would be most important to you?
- Is there anything you'd like to add?
