# Supplementary material for: ‘They will be afraid to touch you’: LGBTI people and sex workers' experiences of accessing healthcare in Zimbabwe—an in-depth qualitative study
Source: BMJ Glob Health. 2017 Apr 13;2(2):e000168. doi: 10.1136/bmjgh-2016-000168 (PMC5435254; doi:10.1136/bmjgh-2016-000168)
Supplement: supplementary appendix [file bmjgh-2016-000168supp_appendix2.pdf]

APPENDIX 2 - INTERVIEW SCHEDULE FOR SEX WORKERS

Date ________________________

Interviewer ________________________

Town/District ________________________

Organization/Facility/Care setting ______________________

Venue ________________________

Demographic information:

Tribe ______________________

Interviewee name ______________________

Age ______________________

Gender ______________________

Sexual identity ______________________

Relationship status ______________________

Primary diagnosis ______________________

Time since diagnosis ______________________

Exploration of health services:

- As someone who identifies as a sex worker, in what ways do you think your experience of your illness might be different to people who do not identify as sex workers?
- How about your experience of care, how might that have been different?
- When you are with your health care providers, (doctors, nurses, and other members of the health care team) do any of them know of your work?
  - YES….how?
  - NO….why?
- Is this how you prefer it to be?
- How do you feel about being asked directly about your sex work?
- Should you be asked directly by the health care providers, or should it come from you first?
- What kinds of things make it easier/less easy to let health care providers know of your sex work? (PROMPT: communicative, environmental, societal, institutional)
- Has the gender of the health care provider been relevant in disclosing your work?

Communication and Sex Work:

- When taking your personal history or talking to you during appointments, do health care providers ever refer to your sex work? How do they do it….WELL/BADLY?
- Do you feel they respect and understand your work? How do they demonstrate this?
- Are there phrases or words that the health care providers have used that made you feel more able to talk about your work?
- How about in terms of the way they talk to you – their manner, body language?
- Do you find the health care providers use the same words to describe sex work as you?
- Can you tell me about when it hasn't been handled well?
- Are there phrases or words used by the health care providers that made you feel uncomfortable about sharing your sex work?
- Have there been times when their manner or body language has stopped you sharing your work? How?
- How would you like your sex work to be acknowledged and referred to by the health care providers?

Involvement of Partner: (IF THEY HAVE PARTNER/EQUIVALENT)

- Has your partner or family attended appointments with you?
- Do you want them present?
- Have you felt they would be welcome?
- Can you tell me how they are acknowledged by health care providers?
- Have they talked about what the experience was like for them?
- Would you like it to be any different? How?
- Do you feel your partner/family have enough support?
- What forms of additional support would be useful for them?

Support Structures:

- Where do you get your support from? (PROMPT: partner, family, communities, institutional)
- Are they known to your health care team? How/why?

- Who would you say are your main sources of care? (outside of professional services like doctors nurses and social worker). (PROMPT: CAREFULLY PROBE FOR BIOLOGICAL VS CHOSEN FAMILY AND NETWORKS)

For those with current serious Illness: illness history:

- Can you tell me something about your illness? When did you first become ill?
- Can you tell me where you've received care for your illness? (e.g. Primary, secondary, tertiary care)
- How do you learn about updated developments in your illness and its treatment?
- What are your main needs and problems? (PROMPT: physical, psychological, social spiritual)
- How do health providers help you manage any physical, emotional, social or spiritual problems you or your family or colleagues may encounter from a diagnosis of a life-shortening condition?

Planning for Future Care:

- Looking forward, if your health changes and decisions need to be made about how best to care for you, who would you like to be involved?
- Do you think this will be done? Can you see any problems?
- Have you had this discussion with anyone? Would you like to, and with whom?
- Do you have someone who could arrange this?

Reflections and Recommendations:

- When you discuss you, your life, and what matters to you with your health care providers, do you think your sex work should be part of that discussion? Why? How?
- Since you have had this diagnosis, do you ever feel that you have been treated differently or unfairly because you identify as a sex worker?
- Do you have any questions that you think you'd like to ask your health care providers but don't?
- Are there questions you feel the health care providers don't ask you?

The aim of our study is to help make LGBT, MSM and sex workers know that they and their partner or significant others can expect good care when they have a serious illness, but also to help health care teams to provide better care.

- What should we teach health care providers?
- What things do you think are important for us to tell the sex worker community? (PROMPT: are there barriers or facilitators to care that we need to explore/highlight)
- What do you think would be the best materials / media to share our findings?
- If there was one thing that we could change about your health care, what would be most important to you?
- Is there anything you'd like to add?
