# Supplementary material for: ‘They will be afraid to touch you’: LGBTI people and sex workers' experiences of accessing healthcare in Zimbabwe—an in-depth qualitative study
Source: BMJ Glob Health. 2017 Apr 13;2(2):e000168. doi: 10.1136/bmjgh-2016-000168 (PMC5435254; doi:10.1136/bmjgh-2016-000168)
Supplement: supplementary appendix [file bmjgh-2016-000168supp_appendix4.pdf]

APPENDIX 4- FOCUS GROUP DISCUSSION GUIDE

(SEX WORKERS)

Date ___________________________

Interviewer ___________________________

Town/District ___________________________

Organization/Facility/Care setting _________________________

Venue ___________________________

Demographic information (to be collected on attendance sheet):

Tribe _________________________

Name/Pseudonym _________________________

Age _________________________

Gender _________________________

Sexual identity _________________________

Relationship status _________________________

Primary diagnosis if any _________________________

Time since diagnosis if applicable _________________________

Language(s) ___________________________

Time began ___________________________

Time ended ___________________________

Group introductions:

Please introduce yourselves briefly. We will also pass around an attendance sheet at the end of the discussion to capture your details for our research.

Guiding questions (not all need to be asked)

Illness History:

- In your experience what illnesses do you think are most common with sex workers?
- What are your thoughts on HIV/AIDS as a life-threatening illness? How important is it to you to be tested for HIV? If you have been tested for HIV, what motivated you to be tested? Have you changed any behaviors since? How easy/difficult has that been?
- Can you tell me where members of your community receive care for a serious illness? (e.g. Primary, secondary, tertiary care)
- How do you learn about updated developments in HIV/AIDS and cancers and their treatment?
- How do you access health services to manage cervical cancer checks, STI and OI treatments and/or ART provision?
- For members of your community living with a life-threatening condition what do you think would be their main needs and problems? (PROMPT: physical, psychological, social spiritual)
- What has been your experience of how health providers help manage any physical, emotional, social or spiritual problems that your colleague or their family may encounter from a diagnosis of a life-threatening condition?
- If you or any of the sex worker community was seriously ill and in pain, how would you access pain medications?
- In your experience does your work/lifestyle improve or decrease your chances of accessing good health care?
- Has any health provider ever discussed palliative care with you?

Exploration of health service access:

- As someone who identifies as a sex worker, in what ways do you think your experience of your illness might be different to people who do not identify as sex workers?
- How about your experience of care, how might that have been different?
- When you are with your health care providers, (doctors, nurses, and other members of the health care team) do any of them know your what work you do?

    - YES….how?
    - NO….why?
- Is this how you prefer it to be?
- How do you feel about being asked directly about your sex work?
- Should you be asked directly by the health care providers, or should it come from you first?
- What kinds of things make it easier/less easy to let health care providers know about your sex work? (PROMPT: communicative, environmental, societal, institutional)
- Has the gender of the health care provider been relevant in disclosing your sex work? Do you experience different care from male or female nurses and doctors?

Communication and Sex Work:

- When taking your personal history or talking to you during appointments, do health care providers ever refer to your sex work? How do they do it….WELL/BADLY?
- Do you feel they respect and understand your work? How do they demonstrate this?
- Are there phrases or words that the health care providers have used that made you feel more able to talk about your sex work?
- How about in terms of the way they talk to you – their manner, body language?
- Do you find the health care providers use the same words to describe sex work as you?
- Can you tell me about when the issue of your sex work hasn't been handled well?
- Are there phrases or words used by the health care providers that make you feel uncomfortable about sharing your sex work?
- Have there been times when their manner or body language has stopped you talking about your work? How?
- How would you like your sex work to be acknowledged and referred to by the health care providers?

Involvement of Partner: (IF THEY HAVE PARTNER/EQUIVALENT)

- Has your partner or family attended appointments with you?
- Do you want them present?
- Have you felt they would be welcome?
- Can you tell me how they are acknowledged by health care providers?
- Have they talked about what the experience was like for them?
- Would you like it to be any different? How?
- Do you feel your partner/family have enough support?
- What forms of additional support would be useful for them?

Support Structures:

- When you get ill, or are in pain or your symptoms make life hard, what do you do? Who do you turn to first?
- Where do you get your support from? (PROMPT: partner, family, communities, institutional)
- Are they known to your health care team? How/why?
- Who would you say are your main sources of care? (outside of professional services like doctors nurses and social worker). (PROMPT: CAREFULLY PROBE FOR BIOLOGICAL VS CHOSEN FAMILY AND NETWORKS)

Planning for Future Care:

- Looking forward, if your health changes and decisions need to be made about how best to care for you, who would you like to be involved?
- If you were to be diagnosed with a life-threatening illness such as cancer, what health care and support do you think you could expect to receive for the rest of your life?
- If you were to reach the end of your life due to a serious illness, what you want to happen?
- Do you think this will be done? Can you see any problems?
- Have you had this discussion with anyone? Would you like to, and with whom?
- Do you have someone who could arrange this?

Reflections and Recommendations:

- When you discuss you, your life, and what matters to you with your health care providers, do you think your sex work should be part of that discussion? Why? How?
- Do you have any questions that you think you'd like to ask your health care providers but don't?
- Are there questions you feel the health care providers don't ask you?
- In your experience how does your sex work affect your access to health care?
- Has any health provider ever discussed palliative care with you?

The aim of our study is to help make sex workers know that they and their partner or significant others can expect good care when they have a serious illness, but also to help health care teams to provide better care.

- What should we teach health care providers?
- What things do you think are important for us to tell the sex worker community about living with life-threatening illness? (PROMPT: are there barriers or facilitators to care that we need to explore/highlight)
- What do you think would be the best materials / media to share our findings?
- If there was one thing that we could change, what would be most important to you?
- Is there anything you'd like to add?
